# Factors Associated with Severe Postoperative Complications (Clavien–Dindo Grades III–IV) After Left-Sided Colon Cancer Surgery: A Single-Centre Retrospective Cohort Study

**DOI:** 10.3390/life16091528

**Published:** 2026-09-14

**Authors:** Claudiu Daha, Hortensia-Alina Moisă, Ciprian Cirimbei, Șerban Andrei Marinescu, Mihnea Alecu, Laurențiu Simion

**Affiliations:** 1 Clinical Department No 10, General Surgery, University of Medicine and Pharmacy “Carol Davila”, 050474 Bucharest, Romania; claudiu.daha@umfcd.ro (C.D.); ciprian.cirimbei@umfcd.ro (C.C.); serban.marinescu@yahoo.com (Ș.A.M.); mihneaalecu@yahoo.com (M.A.); lasimion@yahoo.com (L.S.); 2 Department I of Oncological Surgery, Oncological Institute “Prof. Dr. Alexandru Trestioreanu”, 022328 Bucharest, Romania

**Keywords:** left-sided colon cancer, postoperative complications, risk factors, anastomotic leakage, short-term outcomes

## Abstract

Postoperative morbidity remains a significant challenge in the surgical treatment of colorectal cancer. Nevertheless, studies specifically evaluating short-term postoperative morbidity following left-sided colon cancer surgery are limited, and the factors associated with adverse postoperative outcomes have not yet been fully characterised. This study investigates factors associated with severe postoperative complications following left-sided colon cancer surgery. A retrospective, single-centre cohort study was conducted, including 234 consecutive adult patients who underwent curative or palliative surgery for histologically confirmed left-sided colon cancer between January 2018 and December 2024. Demographic, clinicopathological, laboratory, and surgical variables were collected. Univariate binomial logistic regression and Firth-penalised multivariable logistic regression were performed to identify factors associated with severe postoperative complications (Clavien–Dindo grades III–IV). Left-sided colon cancer frequently presented at advanced stages or with complicated forms, with severe postoperative morbidity occurring in 9.0% of patients. Anastomotic leakage occurred in 8 of 153 patients with anastomoses (5.2%), with an associated overall mortality of 1.3%. In our cohort, lower preoperative haemoglobin levels, lower early postoperative albumin and total protein values, larger tumours, and synchronous colorectal tumours were associated with higher odds of severe postoperative complications. Our findings provide additional evidence on the incompletely characterised morbidity following left-sided colon cancer surgery.

## 1. Introduction

The recognition of colon cancer as an entity distinct from rectal cancer in contemporary guidelines [1,2] has given rise to growing evidence highlighting the biological and clinical heterogeneity of colon cancer itself. Consequently, right- and left-sided colon cancers are increasingly regarded as separate clinicopathological entities, differing in embryological origin, vascular anatomy [3], molecular profile [4], tumour microenvironment, surgical techniques [5], response to systemic therapies, and oncological outcomes [6,7,8,9,10].

These distinctions may also influence surgical management and postoperative results, supporting the need for studies focused on anatomically defined subgroups rather than colon cancer as a single homogeneous disease. Tumours located in the left colon (between the distal third of the transverse colon and the rectosigmoid junction) may require different surgical resections, including left/-extended hemicolectomy, splenic flexure colectomy, sigmoidectomy, rectosigmoid resection, or, in very rare cases, sub/-total colectomy. A primary anastomosis is the preferred option when it can be performed safely and there is no indication for a Hartmann procedure [11]. In the context of surgical decision-making, the vascular supply to the left colon assumes particular significance, given the presence of watershed areas such as Griffiths’ and Sudeck’s points [12,13]. The roles played by the inferior mesenteric and left colic arteries [14]; splenic flexure mobilisation; and the necessity of achieving a safe, tension-free, and well-perfused anastomosis are pivotal elements in this regard. These particularities may influence anastomotic healing and postoperative morbidity, placing anastomotic leakage among the major contributors to Clavien–Dindo III–IV complications [15]. The reported risk of leakage varies considerably between studies, depending on tumour location (ranging from 5.1% following sigmoid surgery to 10.7% following rectal surgery) [16]. Despite the extensive research conducted on colorectal cancer surgery, the majority of studies evaluate both colon and rectal cancers or combine right- and left-sided colon tumours [17,18,19,20]. This methodological approach may dilute the specific risk profile associated with left-sided colon cancer surgery.

A number of risk factors have been delineated, including male gender, elevated ASA (American Society of Anesthesiologists) score, the presence of comorbidities [16], anaemia, hypoalbuminemia, malnutrition [21], systemic inflammation, prolonged operative time, substantial blood loss, blood transfusion, more distal anastomosis (colorectal), and technical factors related to perfusion and tension [22,23,24]. It is widely acknowledged that nutritional and inflammatory statuses are significant contributors to postoperative outcomes [25]. Hypoalbuminemia has been demonstrated to be associated with impaired wound healing and an increased risk of anastomotic leakage, while anaemia has been shown to reduce tissue oxygenation [26,27]. Furthermore, leucocytosis, neutrophilia, and inflammatory haematological ratios (e.g., neutrophil-to-lymphocyte ratio) have been investigated as markers of postoperative septic complications and severe morbidity. Measuring these biological parameters before or shortly after surgery facilitates identification of patients who are at increased risk [28,29,30,31].

Notwithstanding the prolonged adenoma–carcinoma sequence that characterises colon carcinogenesis and provides a substantial opportunity for early detection [32,33], a significant proportion of patients are still diagnosed with locally advanced or metastatic disease. Consequently, open surgery remains indispensable in the management of locally advanced colon cancer (LACC) requiring complex multiorgan oncological resection, with rates of up to 19.2% in different studies [34]. Its role is also important in palliative settings, wherein the aim is to control symptoms and prevent complications related to the disease.

In addition, a significant subset of patients with left-sided colon cancer present with acute tumour-related complications, usually large bowel obstruction, followed by haemorrhage and septic manifestations or intricate forms. Such presentations frequently necessitate urgent surgical intervention and are associated with increased perioperative risk and a reduced likelihood that minimally invasive treatment will be possible. For patients with stenosing tumours, a complete preoperative assessment of the colon is often unfeasible, limiting the accuracy with which the extent of the disease can be characterised and increasing the probability of encountering unexpected intra- or postoperative findings. Moreover, the anatomical localisation of colonic lesions established by preoperative imaging and endoscopy does not always entirely match intraoperative findings. The increasing prevalence of multiple colorectal neoplasms, both synchronous and metachronous, further complicates surgical decision-making [35]. Management of these lesions may require extended colectomies, multi-segmental resections, or staged procedures, necessitating a careful balance between oncological radicality and the preservation of postoperative bowel function.

Tumours located at the splenic flexure pose specific surgical challenges owing to their proximity to the spleen and pancreas [36]. Concerning sigmoid tumour location, the presence of a dolichosigmoid facilitates adequate mobilisation and allows segmental oncological resections while maintaining favourable conditions for tension-free reconstruction. Conversely, this configuration makes one susceptible to developing inflammatory or neoplastic adhesions on pelviabdominal structures (ranging from direct invasion of adjacent organs to extension beyond the conventional surgical boundaries, most commonly the urinary bladder, uterus, or adnexa). Hartmann’s procedure is considered a safer alternative in high-risk patients or emergency settings, but it still has its own spectrum of postoperative adverse events.

In view of the distinct clinical and surgical characteristics of left-sided colon cancer and the paucity of evidence specifically addressing this patient population, in this study, we aimed to evaluate the factors associated with severe short-term postoperative complications following surgery for left-sided colon cancer at a dedicated oncology centre, thereby contributing additional evidence to the existing literature, allowing comparison with previously reported findings, and identifying areas for future research.

## 2. Materials and Methods

### 2.1. Study Design and Patient Selection

A retrospective, observational, single-centre, multi-surgeon study was conducted at the Department I of General Surgery, Institute of Oncology “Prof. Dr. Al. Trestioreanu”, Bucharest, Romania. A total of 234 consecutive adult patients who had undergone primary surgery with either curative or palliative intent for histologically confirmed left-sided colon cancer over a 7-year period (from January 2018 to December 2024) were included. Left-sided colon cancer was defined as tumours arising from the distal third of the transverse colon, the splenic flexure, the descending colon, the sigmoid colon, and the rectosigmoid junction. Patients presenting with secondary involvement of the left colon due to direct invasion from other primary malignancies (e.g., retroperitoneal sarcoma, ovarian cancer, or carcinomatosis of unknown primary origin) or with metastatic lesions involving the colon originating from other primary tumours (e.g., breast cancer) were excluded from the study.

### 2.2. Data Collection

Clinical data were retrospectively collected mainly from medical records, institutional hospital electronic databases, operative reports, pathology reports, and documentation from all inpatient and outpatient visits, including consultations in the Departments of Medical Oncology and Radiation Oncology. Any uncertainties regarding data collection were resolved through discussion with the treating physicians.

The following variables were collected for this study: demographic characteristics (age, sex, and area of residence); comorbidities; preoperative laboratory parameters (baseline haematological and fibrinogen values, from which neutrophil-to-lymphocyte ratio (NLR) and platelet-to-lymphocyte ratio (PLR) indices were calculated); American Society of Anesthesiologists (ASA) score; surgical variables (operative approach, type of resection, type of anastomosis, multi-visceral resection, operative time, and other technical aspects); tumour-related characteristics (location, size, histology, grade, p TNM stage, and other pathological findings); postoperative day 1–3 (POD 1–3) serum proteins (albumin and total proteins) and serum C-reactive protein (CRP) values; distant metastases; and postoperative outcomes.

### 2.3. Outcome Measures

The primary outcome of this study was the occurrence of severe postoperative complications within 30 days after surgery. The secondary outcomes included the incidence of anastomotic leakage, postoperative mortality, length of postoperative stay, and identification of factors associated with severe postoperative morbidity. Postoperative complications occurring within 30 postoperative days were graded according to the Clavien–Dindo (CD) classification based on the therapeutic intervention required [37,38]. For patients with multiple postoperative complications, the highest grade was considered for statistical analysis. Postoperative mortality was analysed and reported separately. Anastomotic leakage (AL) was defined, in accordance with the criteria established by the International Study Group of Rectal Cancer (ISREC), as a disruption in the integrity of the intestinal wall at the site of the colorectal or colo-colic anastomosis, resulting in “communication between the intra- and extraluminal compartments”, and it was further graded as A, B, or C [39]. The median follow-up period was 386 days, while the mean was 610.6 days (range: 0–2906 days).

Statistical analyses were performed using R software ((version 4.6.0) The R Foundation for Statistical Computing, Vienna, Austria) [40]. A descriptive analysis was performed in order to compare patients who developed anastomotic leakage with those who did not. Continuous variables were summarised using the mean, standard deviation (SD), median, interquartile range (IQR), and minimum and maximum values. Categorical variables were summarised as absolute (*n*) and relative (%) frequencies. Critically, complete data were not available for all patients, and no imputation methods were used to address missing data. The Clavien–Dindo grades III–IV complications and anastomotic leakage were analysed separately as binary outcomes (yes/no). A two-sided significance level (alpha, α) was set, namely, 0.05, and *p*-values < 0.05 were considered statistically significant. Univariate binomial logistic regression analysis was performed to evaluate the association between each potential predictor and the occurrence of severe postoperative complications and, separately, anastomotic leakage. The dependent variable in this study was the presence or absence of Clavien–Dindo grades ≥ III complications or anastomotic leakage, while the independent variables included demographic, clinical, pathological, laboratory, and perioperative variables collected in this study. The results obtained are presented as odds ratios (ORs) with 95% confidence intervals (95% CIs).

Given the limited number of outcome events, Firth-penalised multivariable logistic regression was used to reduce small-sample bias [41], using a limited number of clinically relevant variables. Results were reported as adjusted odds ratios (ORs), 95% confidence intervals (CIs), and *p*-values.

### 2.4. Ethical Consideration

The study was conducted in accordance with the principles of the Declaration of Helsinki and approved by the Institutional Ethics Committee of the Institute of Oncology “Prof. Dr. Al. Trestioreanu”, Bucharest (Protocol number 14685/Date of approval 22 July 2026). Informed consent from individual patients was considered unnecessary given the retrospective nature of our study. Even so, all patients admitted for treatment in our institution sign a general consent form permitting anonymous usage of medical data and results for clinical research, educational purposes, and developing and publishing scientific publications.

## 3. Results

The cohort comprised 234 patients, the majority of whom (73.1%) were over 60 years of age, with the highest incidence rate observed in the 61–70 age group. We observed a modest predominance of male patients, who accounted for 53.8% of the population, and nearly two-thirds of patients (65.8%) resided in urban areas. In total, 16.2% of the patients had a previous history of malignancy. The most prevalent comorbidity was cardiovascular disease, accounting for 65.0% of cases. Overall, 89.3% of patients initially presented with complicated left-sided colon cancer, mostly with stenosing tumour (70.9%) and haemorrhagic disease (56.4%). For the purposes of this study, complicated colon cancer was operationally defined as the presence of one or more of the following clinical presentations: a stenosing tumour (characterised by preoperative complete or incomplete colonic stenosis, confirmed histopathologically as luminal narrowing involving ≥50% of the bowel circumference); haemorrhagic disease (encompassing tumour-related gastrointestinal bleeding, including haematochezia, rectorrhagia, or occult blood loss and/or anaemia with a haemoglobin level < 12 g/dL, according to the institutional laboratory reference range); and septic disease (defined by leucocytosis > 11,000/uL, according to the institutional laboratory reference range, and/or tumour perforation, localised or generalised peritonitis, or a peritumoral abscess). Notably, none of the patients underwent neoadjuvant chemotherapy or radiotherapy for the index colon cancer. However, a subset of the patients had undergone chemotherapy and/or radiotherapy for previous malignancy, including six patients with metachronous colorectal cancer. Preoperative endoscopic stenting as a bridge to surgery was not performed in patients with stenosing left-sided colon cancer included in this cohort (Table 1).

With regard to pathological stage, stage I disease was identified in 33 patients (14.1%). Conversely, stage IV disease was found in 74 patients (31.6%). Among the 74 patients with stage IV disease, the metastatic pattern spread consisted of a single distant organ in 44 patients (59.4%), two distant organs in 13 (17.6%), and peritoneal metastases in 17 (23.0%). Synchronous surgical treatment of liver metastases was performed in 6 patients (8.1%)—from a total of 57 patients—including hepatic metastasectomy, non-anatomical liver resection, radiofrequency ablation (RFA), or a combination of these techniques. Stages II and III accounted for 70 (29.9%) and 57 (24.4%) patients, respectively.

### 3.1. Surgical Characteristics

Rectosigmoidectomy was the most commonly performed procedure (124 patients, 53.0%), followed by segmental colectomy (68 patients, 29.1%), which included 45 sigmoid and 14 splenic flexure colectomies. Left or extended left hemicolectomy accounted for 23 patients (9.8%), whereas subtotal colectomy and pelvic exenteration were required in one (0.4%) and two female patients (0.9%), respectively. Colostomy or other bowel diversion procedures were performed in 14 patients (6.0%).

Multiorgan resection was performed in 21 patients (9.0%). The most frequently resected organs were the small bowel (segmental enterectomy, n = 8) and the adnexa (unilateral or bilateral adnexectomy, n = 8), followed by the urinary bladder (partial cystectomy, n = 6) and the spleen (splenectomy, n = 6). The other multi-visceral resections included distal splenopancreatectomy (n = 2), abdominal wall/peritoneal resection (n = 2), total hysterectomy with bilateral adnexectomy (n = 1), and ileocecal resection (n = 1). As some patients required en bloc resection of multiple adjacent organs, the total number of organ resections clearly exceeded the number of patients undergoing multi-visceral surgery.

In terms of surgical approach, the majority of patients underwent open surgery (200, 85.5%), while laparoscopic surgery was performed in 34 patients (14.5%), with a conversion rate of 17.6%. The most frequent reasons for conversion were uncontrolled intraoperative bleeding, primarily due to splenic injuries, extensive intra-abdominal adhesions, and tumours with greater-than-anticipated extension identified during surgical exploration.

A total of 55 postoperative complications were recorded. According to the Clavien–Dindo classification system, five complications (9.1%) were categorised as grade I, twenty-nine (52.7%) were categorised as grade II, six (10.9%) were categorised as grade IIIa, eight (14.5%) were categorised as grade IIIb, five (9.1%) were categorised as grade IVa, and two (3.6%) were categorised as grade IVb. Severe complications Clavien–Dindo grades III–IV) occurred in 21 patients (9%). Of these patients, 19 experienced severe surgical complications, and 8 experienced severe medical complications, with some patients developing more than one severe complication.

The surgical complications encountered included anastomotic leakage (n = 6), abdominal wall complications requiring reoperation (wound infection and/or complete/incomplete dehiscence, n = 5), postoperative haemorrhage requiring surgical intervention (n = 3), stoma dehiscence (n = 2), rectal stump necrosis (n = 1), postoperative pancreatic fistula and acute pancreatitis (n = 1), and postoperative bladder paresis following partial cystectomy (n = 1). The severe medical complications comprised pulmonary complications requiring admission to the intensive care unit (ICU) (n = 5), combined hepatorenal failure (n = 1), acute renal failure (n = 1), and acute lower-limb ischaemia (n = 1).

A primary intestinal anastomosis was employed in 153 patients (65.4%), for which the following protective measures were used: diverting ileostomy (n = 5), cecostomy (n = 3), and transanal transanastomotic drainage (n = 3). Nevertheless, anastomotic leakage occurred in eight patients (5.2%). Six of these instances occurred after a colo-colic anastomosis was established, and two occurred after a colorectal anastomosis was created. Leakage was diagnosed between postoperative days 3 and 15. Two patients were managed conservatively (AL grade B), whereas six required reoperation (AL grade C). Three of these patients died following reoperation because of septic complications resulting from anastomotic leakage, with an overall associated mortality of 1.3%. The anastomotic leakage rates were 3.57% (n = 1) following laparoscopic surgery and 5.6% (n = 7) following open surgery.

### 3.2. Primary Outcome—Factors Associated with Severe Postoperative Complications (Clavien–Dindo Grades III–IV)

No significant associations between sex, age, residential area, and severe postoperative complications were found.

Of the 234 patients included in the study, 17 (7.3%) were diagnosed with synchronous colorectal cancer, 9 (3.8%) were diagnosed with metachronous colorectal cancer, and 4 (1.7%) underwent surgery for anastomotic recurrence. The presence of synchronous tumours was associated with 3.62-fold higher odds of severe postoperative complications (OR 3.62; 95% CI 0.94–11.6; *p* = 0.040). Additionally, greater tumour size (measured as the largest tumour dimension in the pathological report) was significantly associated with a higher likelihood of severe postoperative complications. Each 1 cm increase in tumour size was associated with a 23% higher probability of Clavien–Dindo grades III–IV complications (OR 1.23; 95% CI 1.04–1.47; *p* = 0.016).

No statistically significant associations were observed between severe postoperative complications and pathological characteristics (including pathological T, N, or M stage, grade, lymphovascular invasion, peritumoral inflammatory infiltrate, or tumour budding (all *p* > 0.05)).

Among the preoperative haematological and inflammatory parameters, only haemoglobin was significantly associated with severe postoperative complications. Each 1 g/dL increase in preoperative haemoglobin was associated with a 19% reduction in the odds of Clavien–Dindo grades III–IV complications (OR, 0.81; 95% CI, 0.66–0.98; *p* = 0.033). Anaemia (OR, 2.33; 95% CI, 0.94–6.12; *p* = 0.072) and the neutrophil-to-lymphocyte ratio (OR, 1.11; 95% CI, 0.98–1.25; *p* = 0.082) showed a trend toward an increased risk, but it did not reach statistical significance. No significant associations were observed for the remaining haematological variables.

Analysis of the postoperative laboratory parameters measured on days 1–3 (POD 1–3) revealed that higher total protein and albumin values were significantly associated with a lower likelihood of severe postoperative complications. Each 1 g/dL increase in total proteins was associated with a 57% lower probability of Clavien–Dindo grades III–IV complications (OR, 0.43; 95% CI, 0.22–0.77; *p* = 0.009), while each 1 g/dL increase in albumin was associated with an 87% lower probability of severe postoperative complications (OR, 0.13; 95% CI, 0.03–0.40; *p* < 0.001). In contrast, postoperative C-reactive protein (CRP) levels were not significantly associated with severe postoperative complications (OR, 1.06; 95% CI, 0.98–1.15; *p* = 0.12).

Neither ASA score, surgical approach, anastomotic configuration, nor operative duration was significantly associated with severe postoperative complications. Although patients undergoing open surgery had higher odds of experiencing Clavien–Dindo grades III–IV complications than those treated laparoscopically (OR, 2.90; 95% CI, 0.57–53.1), this association did not reach statistical significance (*p* = 0.31) (Table 2).

Given the limited number of events, Firth-penalised multivariable logistic regression was performed for the primary outcome using a limited number of clinically selected variables. The analysis included 101 patients with complete data. In the adjusted model, tumour diameter was independently associated with Clavien–Dindo grades ≥ III complications (OR, 1.30; 95% CI, 1.01–1.75; *p* = 0.044). Postoperative albumin showed an inverse association with the outcome, although this association did not reach statistical significance after adjustment (OR, 0.36; 95% CI, 0.09–1.09; *p* = 0.073) (Table 3).

### 3.3. Secondary Outcome—Factors Associated with Anastomotic Leakage

Patients with anastomotic leakage had substantially longer postoperative hospital stays than those without anastomotic leakage (mean: 31 ± 13 vs. 10 ± 5 days; median: 29 vs. 8 days).

Microscopically positive (R1) resection margins were identified in only 1% of patients who were treated using a primary anastomosis, and none of these patients developed anastomotic leakage (Table 4).

Given the major impact of anastomotic leakage on the early postoperative course, a separate analysis was performed to identify factors associated with its occurrence.

Higher preoperative haemoglobin levels were significantly associated with lower odds of anastomotic leakage. For every 1 g/dL increase in haemoglobin, the odds of anastomotic leakage were reduced by 34% (OR, 0.66; 95% CI, 0.46–0.92; *p* = 0.018).

Higher early postoperative albumin levels were significantly associated with lower odds of developing anastomotic leakage. Early postoperative albumin concentration was defined as the first available serum albumin measurement obtained on postoperative days 1–3 (POD 1–3). Each 1 g/dL increase in albumin levels was associated with a 97% reduction in the odds of leakage (OR, 0.03; 95% CI, 0.001–0.27; *p* = 0.011). Postoperative total protein levels also showed an inverse association with the odds of anastomotic leakage (OR, 0.53; 95% CI, 0.19–1.20), although this association did not reach statistical significance (*p* = 0.16). This analysis was restricted to this subgroup because serum albumin and total protein levels during the first three postoperative days were available only for these patients.

The number of lymph nodes harvested was significantly associated with the occurrence of anastomotic leakage. For each additional lymph node retrieved, the odds of developing anastomotic leakage fell by 14% (OR, 0.86; 95% CI, 0.74–0.98; *p* = 0.038).

Colorectal anastomoses were associated with approximately 4.5-fold lower odds of anastomotic leakage than colo-colic anastomoses (OR, 0.22; 95% CI, 0.03–0.97; *p* = 0.044). Operative duration was not significantly associated with the odds of anastomotic leakage (OR, 0.99; 95% CI, 0.98–1.00; *p* = 0.15) (Table 5).

## 4. Discussion

Unlike many previous studies, which grouped colon and rectal cancers together for analysis, our study focuses exclusively on surgery for left-sided colon cancer and provides a thorough evaluation of early postoperative outcomes in a large cohort from a single centre. Given the complexity of the cohort, the overall incidence of severe complications is in line with that reported in similar studies, with anastomotic leakage being the most significant complication because of its impact on postoperative recovery, the need for further interventions, prolonged hospitalisation, and mortality after surgery. With a leakage rate of 5.2%, our results are consistent with those reported in the recent literature on left-sided colorectal resections, wherein incidences generally vary between 5.1% and 7.9% [16,42]. Similarly, Kryzauskas et al. reported an anastomotic leakage rate of 5.1% following sigmoid resection, which is comparable to the rate of 5.2% observed in our cohort [16]. Among the few studies focusing exclusively on left-sided colon cancer, Pellino et al. reported an anastomotic leakage rate of 7.9% and a perioperative mortality rate of 2.0% in patients undergoing curative left colectomy with a primary stapled anastomosis [42]. Despite including both curative and palliative procedures, as well as both stapled and hand-sewn anastomoses, our cohort had slightly lower rates of anastomotic leakage and postoperative mortality (1.3%). In both studies, anastomotic leakage was a major contributor to postoperative mortality.

The high proportion of open surgical procedures in our study is likely due to the advanced stage and complexity of the disease rather than limited expertise in laparoscopic surgery. Many patients required extensive resections, management of locally advanced tumours, or emergency procedures—situations in which an open approach is preferred. The lower leakage rate observed in the laparoscopic group is likely secondary to differences in patient selection and case complexity rather than the surgical approach itself. Although our conversion rate was 17.6%, this method should be interpreted as a safety-first approach that prioritises patient safety and oncological adequacy. As Horesh et al. previously reported, conversion should not be viewed as surgical failure, as they demonstrated that conversion from minimally invasive to open surgery was associated with similar short-term mortality and improved overall survival compared with upfront open surgery, particularly in patients with left-sided colon cancer, supporting the prudence of employing an initial minimally invasive approach whenever technically feasible [43].

Our analysis identified several perioperative variables associated with severe postoperative complications.

Patients with milder anaemia were found to be at a reduced risk of postoperative morbidity. Although the correlation with anaemia only achieved borderline statistical significance, this tendency is consistent with previous reports [44]. Therefore, preoperative haemoglobin levels may represent a potentially modifiable factor associated with postoperative outcomes, although the potential benefit of its optimisation requires prospective validation.

Early postoperative total serum protein and albumin levels were associated with severe postoperative complications, suggesting that these parameters may serve as early postoperative markers of adverse outcomes. Unfortunately, in our study, this analysis was performed on a small subgroup of just 68 patients, of whom only 5 experienced anastomotic leakage, a factor that might have introduced selection bias. Consequently, although the association was statistically significant (OR, 0.03; 95% CI, 0.001–0.27; *p* = 0.011) because of the small number of events, these findings should be considered exploratory and require confirmation in larger, adequately powered cohorts. In a retrospective cohort study of 403 patients aged 80 years or over undergoing curative resection for colon cancer (excluding rectal cancer), Niu et al. demonstrated that preoperative serum albumin levels below 35 g/L and right-sided tumour location were independent predictors of severe postoperative complications (Clavien–Dindo grades III–V) [45,46].

The CoReAL (The Consensus for Reporting of Colorectal Anastomotic Leaks) international consensus on reporting anastomotic leakage after colorectal cancer surgery identified low preoperative serum albumin levels and tumour size greater than 5 cm as modifiable risk factors for anastomotic leakage [47,48]. Our findings further reinforce these recommendations, as larger tumours were also associated with an increased likelihood of severe complications, possibly reflecting more technically demanding surgery with greater tissue dissection, and an increased risk of adjacent organ involvement.

Systemic inflammatory status also appeared to influence postoperative outcomes. In a recent retrospective study, Laitakari et al. demonstrated that a preoperative NLR of at least 4.0 independently predicted severe postoperative complications (Clavien–Dindo grades IV–V) following curative colon cancer resection (OR, 3.48; 95% CI, 1.57–7.75) [24]. In contrast, although higher NLR values in our cohort indicated a trend towards a greater risk of severe postoperative complications, this association did not reach statistical significance (OR, 1.11; 95% CI, 0.98–1.25; *p* = 0.082). However, direct comparison between studies is limited because their cohort included both right- and left-sided colon cancers (37.9%). In contrast with previous studies [49], CRP was not associated with severe complications. This may partly reflect its postoperative assessment in a limited subset of our patients, representing an additional limitation of our study.

Furthermore, synchronous colorectal tumours were associated with increased postoperative morbidity. This is a particularly relevant observation in our study because synchronous tumours often require more extensive resections and tailored reconstructive strategies, thereby increasing the complexity of the operation. An increasing amount of attention is being directed towards the molecular biology and genetic profiles of these tumours, which are known to influence patient outcomes beyond surgery [50]. Relatively few studies have investigated the impact of synchronous tumours on short-term postoperative outcomes specifically, making this one of the more original observations of our analysis.

Regarding multi-visceral resections for locally advanced colon cancer, a comparative study including 203 patients undergoing either standard colectomy or multi-visceral colectomy reported anastomotic leakage rates of 4.5 and 5.5%, respectively, and a postoperative mortality rate of 3.3%. In their study, a tumour being located on the left side of the colon was associated with postoperative morbidity amounting to Clavien–Dindo grades ≥ II [34].

An unexpected finding of our study was the inverse relationship between the number of lymph nodes harvested and the likelihood of anastomotic leakage occurring. For each additional lymph node retrieved, the odds of leakage were reduced by 14%. A higher lymph node yield may reflect more extensive, standardised anatomical resections rather than a direct protective effect on anastomotic healing.

The lower odds of anastomotic leakage for colorectal compared with colo-colic anastomosis in the univariate analysis were based on only two leakage events, making the estimate unstable.

Other variables expected to influence postoperative morbidity, such as ASA score and operative duration, were not significantly associated with severe complications, possibly reflecting the limited number of severe events in our cohort.

In the Firth-penalised multivariable analysis, tumour diameter remained independently associated with severe postoperative complications, with each 1 cm increase corresponding to a 30% higher probability of a Clavien–Dindo III-IV outcome. Early postoperative albumin showed an inverse association, although this association did not reach statistical significance after adjustment. These findings should be considered preliminary given the limited number of events and the small complete-case sample.

This study has several important strengths. It includes a broad range of clinical presentations and disease stages, enhancing its applicability in routine oncological surgical practice. Our findings add to the limited body of evidence focusing exclusively on postoperative complications following left-sided colon cancer surgery, an area that remains underrepresented in the literature.

Nevertheless, this study also has several limitations. Its retrospective observational design introduces the possibility of selection bias, and its single-centre nature may limit its generalisability. The study period encompassed the period during which the restrictions imposed by the pandemic (January 2020–May 2023) limited patients’ access to diagnostic investigations, treatment, and postoperative follow-ups. This potential discrepancy could have influenced patient management and the completeness of the data. The COVID-19 pandemic caused by SARS-CoV-2 may have contributed to the relatively low number of surgical procedures performed for this condition and the high proportion of patients presenting with advanced-stage disease and multiple tumours, likely reflecting delays in diagnosis, referral, and access to surgical care. Importantly, certain laboratory parameters were unavailable for all patients, so there was a smaller cohort of patients for certain laboratory values. Furthermore, the relatively low number of anastomotic leakage events reduced statistical power. The involvement of multiple surgeons is a limitation because of variability in surgical technique, although all procedures were performed by surgeons experienced in colon cancer surgery.

Surgery performed to the highest standards is defined not only by oncological radicality but also by the minimisation of postoperative morbidity and the preservation of quality of life. Severe postoperative complications, 30-day mortality, unplanned readmissions, and hospital stay are widely accepted indicators of surgical quality [51,52]. These factors influence postoperative recovery, the timely initiation of adjuvant therapy, healthcare resource utilisation, and, ultimately, long-term oncological outcomes [53,54].

The significance of our findings is twofold: firstly, several of the risk factors identified are manageable prior to surgery and, secondly, because of the potential implications for future research in this field. For instance, careful planning of surgery for patients with large or synchronous tumours may help reduce postoperative morbidity.

Age, sex, and residential area were not significantly associated with severe postoperative complications in our cohort, with age remaining non-significant after adjustment. Recent evidence suggests that postoperative risk in older patients may be more closely related to comorbidity and surgical factors than to chronological age alone. The predominance of urban patients may also have limited our ability to identify differences according to residential setting [55,56].

## 5. Conclusions

Left-sided colon cancer is often diagnosed at an advanced stage or with complicated forms, resulting in considerable postoperative morbidity.

In this study, anastomotic leakage was the most significant surgical complication, with an incidence similar to that reported in the contemporary literature.

Several perioperative factors showed associations with severe postoperative complications, including haemoglobin levels, tumour size, synchronous colorectal tumours, and early postoperative serum albumin and total protein levels. Given this study’s observational nature and the limitations of the available data, these findings should be interpreted as exploratory associations rather than evidence of independent predictive or causal relationships.

Despite its retrospective, single-centre design, this study provides additional evidence on postoperative morbidity following left-sided colon cancer surgery.

Further multicentre prospective studies must be conducted to validate these findings and explore whether national development, healthcare resources, access to screening and treatment, and other contextual factors influence postoperative morbidity and mortality following surgery for left-sided colon cancer.

## Figures and Tables

**Table 1:** Baseline demographic and clinical characteristics of patients undergoing surgery for left-sided colon cancer.

Patients’ Characteristics	*n* (%)
Total patients	234 (100)
Age group (years)	
41–50	14 (6.0)
51–60	49 (20.9)
61–70	95 (40.6)
71–80	62 (26.5)
>80	14 (6.0)
Age > 60 years	171 (73.1)
Sex	
Male	126 (53.8)
Female	108 (46.2)
Residence	
Urban	154 (65.8)
Rural	80 (34.2)
Comorbidities *	
Cardiovascular disease	152 (65.0)
Diabetes mellitus	49 (20.9)
Obesity	45 (19.2)
Pulmonary disease	21 (9.0)
Neurological disorders	20 (8.5)
Chronic renal failure	19 (8.1)
Hepatic failure	9 (3.8)
Previous malignancy	38 (16.2)
Clinical presentation	
Complicated/uncomplicated (left-sided colon cancer)	209 (89.3)/25 (10.7)
Stenosing tumour	166 (70.9)
Haemorrhagic disease	132 (56.4)
Septic disease	57 (24.4)
Two concomitant complicated forms **	84 (40.2)
Three concomitant complicated forms **	31 (14.8)
Tumour location	
Splenic flexure	25 (10.7)
Descending colon	18 (7.7)
Sigmoid colon	127 (54.3)
Rectosigmoid junction	64 (27.3)
Histologic type/subtype	
Adenocarcinoma	232 (99.0)
Mucinous adenocarcinoma	18 (7.7)

* Some patients had multiple comorbidities; therefore, percentages may exceed 100%. ** Percentages are calculated among patients with complicated left-sided colon cancer (n = 209).

**Table 2:** Univariable analysis of factors associated with severe postoperative complications (Clavien–Dindo Grades III–IV).

Predictor	N	CD III–IV, *n*	OR (95% CI)	*p*-Value
Demographic characteristics				
Sex				
Female	108	12	Reference	—
Male	126	9	0.62 (0.24–1.51)	0.29
Age	234	21	1.04 (0.99–1.10)	0.11
Residential area				
Rural	80	9	Reference	—
Urban	154	12	0.75 (0.30–1.99)	0.55
Pathological characteristics				
Grade				
G1	26	3	Reference	—
G2–G3	205	17	0.69 (0.21–3.13)	0.58
Gx	3	1	3.83 (0.15–55.2)	0.33
pT stage				
T1	11	1	Reference	—
T2	29	3	1.15 (0.13–24.9)	0.91
T3–T4	182	16	0.96 (0.17–18.3)	0.97
Tx	12	1	0.91 (0.03–25.1)	0.95
pN stage				
N0	117	9	Reference	—
N1	67	7	1.40 (0.48–3.95)	0.52
N2	34	3	1.16 (0.25–4.17)	0.83
Nx	16	2	1.71 (0.25–7.54)	0.52
pM stage				
M0	160	14	Reference	—
M1	74	7	1.09 (0.40–2.75)	0.86
LVI, yes	187	17	1.08 (0.37–2.94)	0.89
Peritumoral inflammatory infiltrate				
No	9	2	Reference	—
Yes	129	9	0.26 (0.05–1.93)	0.13
Tumour budding				
No	18	1	Reference	—
Yes	95	7	1.35 (0.22–26.1)	0.78
Tumour size	218	19	1.23 (1.04–1.47)	0.016
Stenosing tumour, yes	233	21	1.84 (0.65–6.58)	0.29
Synchronous tumour, yes	234	21	3.62 (0.94–11.6)	0.040
Preoperative haematological and inflammatory parameters				
Hb	233	21	0.81 (0.66–0.98)	0.033
Anaemia, yes	233	21	2.33 (0.94–6.12)	0.072
WBC	233	21	1.07 (0.93–1.22)	0.32
Leucocytosis, yes	233	21	1.83 (0.62–4.83)	0.24
PLT	233	21	1.00 (1.00–1.00)	0.34
Neutrophils	232	20	1.11 (0.96–1.27)	0.14
Lymphocytes	232	20	0.55 (0.23–1.17)	0.16
NLR	232	20	1.11 (0.98–1.25)	0.082
PLR	232	20	1.00 (1.00–1.00)	0.13
Fibrinogen	229	21	0.99 (0.57–1.57)	0.96
Postoperative laboratory parameters (POD 1–3)				
Total protein	106	15	0.43 (0.22–0.77)	0.009
Albumin	112	15	0.13 (0.03–0.40)	<0.001
CRP	161	17	1.06 (0.98–1.15)	0.12
ASA score and surgical characteristics				
ASA score	234	21	1.05 (0.57–1.95)	0.87
Surgical approach				
Laparoscopic	28	1	Reference	—
Open	206	20	2.90 (0.57–53.1)	0.31
Anastomotic configuration				
Side-to-side	17	3	Reference	—
Side-to-end/end-to-side	37	2	0.27 (0.03–1.77)	0.17
End-to-end	99	7	0.36 (0.09–1.79)	0.17
Operative duration	233	21	1.00 (1.00–1.01)	0.77

Abbreviations: ASA, American Society of Anesthesiologists Physical Status Classification; CI, confidence interval; CRP, C-reactive protein; CD, Clavien–Dindo; Hb, haemoglobin; LVI, lymphovascular invasion; NLR, neutrophil-to-lymphocyte ratio; OR, odds ratio; PLR, platelet-to-lymphocyte ratio; PLT, platelet count; POD, postoperative day; WBC, white blood cell count.

**Table 3:** Firth-penalised logistic regression of factors associated with Clavien–Dindo Grades ≥ III. The table below presents the initial multivariable model and the corresponding univariable models for the predictors included in the multivariable model.

Predictor	Multivariable Model *N*	OR (95% CI)	*p*-Value	Univariable Model *N*	OR (95% CI)	*p*-Value
Age	101	1.03 (0.97 to 1.11)	0.33	101	1.04 (0.98 to 1.11)	0.21
Postoperative albumin	101	0.36 (0.09 to 1.09)	0.073	101	0.16 (0.04 to 0.47)	0.002
Haemoglobin	101	0.94 (0.70 to 1.26)	0.70	101	0.84 (0.65 to 1.07)	0.16
NLR	101	0.97 (0.76 to 1.20)	0.77	101	1.14 (0.97 to 1.32)	0.09
pTNM stage				101		
I	17	Reference	—		Reference	—
II	30	1.21 (0.17 to 9.71)	0.85		0.78 (0.13 to 5.18)	0.79
III	22	2.21 (0.30 to 18.2)	0.43		1.50 (0.58 to 9.64)	0.62
IV	32	2.49 (0.36 to 21.9)	0.36		1.24 (0.26 to 7.56)	0.79
Anastomosis performed				101		
Yes	67	Reference	—		Reference	—
No	34	0.27 (0.04 to 1.27)	0.10		0.80 (0.22 to 2.53)	0.72
Synchronous tumours				101		
No	91	Reference	—		Reference	—
Yes	10	3.62 (0.58 to 22.5)	0.16		3.26 (0.71 to 12.91)	0.12
Tumour size	101	1.30 (1.01 to 1.75)	0.044	101	1.28 (1.06 to 1.58)	0.009

Abbreviations: CI, confidence interval; OR, odds ratio; NLR, neutrophil-to-lymphocyte ratio; pTNM, pathological tumour–node–metastasis.

**Table 4:** Demographic, pathological, laboratory, and operative characteristics of the patients with a primary anastomosis (N = 153) according to anastomotic leakage status.

Variable	Overall (N = 153)	No AL (N = 145)	AL (N = 8)
Demographic and pathological characteristics			
Sex, *n* (%)—female/male	67 (44)/86 (56)	63 (43)/82 (57)	4 (50)/4 (50)
Age, years—mean (SD); median (IQR); range	65 (9); 66 (12); 41–86	65 (9); 66 (14); 41–86	68 (7); 67 (9); 59–78
Residence, *n* (%)—rural/urban	47 (31)/106 (69)	43 (30)/102 (70)	4 (50)/4 (50)
Histological grade, n (%)—G1/G2/G3	19 (12)/128 (84)/6 (4)	18 (12)/121 (84)/6 (4)	1 (13)/7 (88)/0 (0)
pT stage, *n* (%)—T1/T2/T3/T4	11 (7)/26 (17)/101 (66)/15 (10)	10 (7)/24 (17)/96 (66)/15 (10)	1 (13)/2 (25)/5 (63)/0 (0)
pN stage, *n* (%)—N0/N1/N2	91 (59)/42 (27)/20 (13)	85 (59)/41 (28)/19 (13)	6 (75)/1 (13)/1 (13)
pM stage, *n* (%)—M0/M1	119 (78)/34 (22)	113 (78)/32 (22)	6 (75)/2 (25)
Synchronous tumours, *n* (%)—no/yes	142 (93)/11 (7)	135 (93)/10 (7)	7 (88)/1 (13)
Lymph node yield—mean (SD); median (IQR); range [missing]	14 (8); 12 (10); 0–47 [3]	14 (8); 13 (10); 2–47 [3]	8 (8); 6 (9); 0–20 [0]
Perineural invasion, *n* (%)—no/yes [missing]	87 (70)/37 (30) [29]	82 (69)/37 (31) [26]	5 (100)/0 (0) [3]
Lymphovascular invasion, *n* (%)—no/yes [missing]	83 (62)/51 (38) [19]	79 (62)/49 (38) [17]	4 (67)/2 (33) [2]
Peritumoral inflammatory infiltrate, n (%)—no/yes [missing]	7 (7)/93 (93) [53]	6 (6)/89 (94) [50]	1 (20)/4 (80) [3]
Resection margins, *n* (%)—R0/R1 [missing]	147 (99)/2 (1) [4]	139 (99)/2 (1) [4]	8 (100)/0 (0) [0]
Tumour budding, *n* (%)—absent/present [missing]	15 (18)/70 (82) [68]	14 (17)/69 (83) [62]	1 (50)/1 (50) [6]
Tumour size, cm—mean (SD); median (IQR); range [missing]	4.67 (2.07); 4.20 (2.50); 0.70–13.00 [1]	4.66 (2.04); 4.20 (2.50); 0.70–13.00 [1]	4.93 (2.72); 5.15 (3.63); 1.00–8.60 [0]
Stenosing tumour, *n* (%)—no/yes [missing]	54 (36)/98 (64) [1]	53 (37)/91 (63) [1]	1 (13)/7 (88) [0]
Large bowel obstruction **, n (%)—no/yes [missing]	144 (95)/8 (5.3) [1]	136 (94)/8 (5.6) [1]	8 (100)/0 (0) [0]
Laboratory characteristics			
Haemoglobin, g/dL—mean (SD); median (IQR); range [missing]	12.61 (2.07); 13.00 (3.00); 7.30–17.00 [1]	12.71 (2.03); 13.10 (2.93); 7.30–17.00 [1]	10.83 (2.11); 11.25 (3.30); 7.60–12.90 [0]
Anaemia, *n* (%)—No/Yes [missing]	97 (64)/55 (36) [1]	94 (65)/50 (35) [1]	3 (37)/5 (63) [0]
WBC—mean (SD); median (IQR); range [missing]	7.90 (2.44); 7.44 (2.93); 3.66–21.43 [1]	7.87 (2.44); 7.44 (2.90); 3.66–21.43 [1]	8.33 (2.58); 7.47 (3.72); 5.49–12.56 [0]
Leucocytosis, *n* (%)—no/yes [missing]	138 (91)/14 (9) [1]	132 (92)/12 (8) [1]	6 (75)/2 (25) [0]
Platelets—mean (SD); median (IQR); range [missing]	296 (104); 279 (106); 129–873 [1]	293 (100); 276 (105); 129–873 [1]	349 (148); 292 (172); 160–597 [0]
Neutrophils—mean (SD); median (IQR); range [missing]	5.36 (2.02); 4.93 (2.11); 2.04–17.07 [1]	5.32 (2.01); 4.93 (2.08); 2.04–17.07 [1]	5.93 (2.38); 4.90 (3.81); 3.64–9.33 [0]
Lymphocytes—mean (SD); median (IQR); range [missing]	1.72 (0.64); 1.63 (0.83); 0.49–4.25 [1]	1.73 (0.65); 1.66 (0.82); 0.49–4.25 [1]	1.50 (0.44); 1.37 (0.43); 0.97–2.22 [0]
NLR—mean (SD); median (IQR); range [missing]	3.47 (1.70); 3.03 (1.96); 1.16–11.63 [1]	3.42 (1.67); 3.01 (1.93); 1.16–11.63 [1]	4.21 (2.08); 3.32 (2.24); 2.36–7.74 [0]
Fibrinogen—mean (SD); median (IQR); range [missing]	4.25 (0.79); 4.15 (0.83); 3.19–8.25 [3]	4.24 (0.78); 4.14 (0.81); 3.19–8.25 [3]	4.31 (1.07); 4.23 (1.16); 3.30–6.61 [0]
Postoperative total proteins—mean (SD); median (IQR); range [missing]	5.67 (1.10); 5.50 (1.85); 2.80–8.30 [90]	5.73 (1.12); 5.50 (2.10); 2.80–8.30 [88]	5.05 (0.55); 4.95 (0.93); 4.50–5.70 [2]
Postoperative albumin—mean (SD); median (IQR); range [missing]	3.19 (0.70); 3.10 (0.90); 1.60–4.70 [85]	3.26 (0.67); 3.20 (1.05); 1.60–4.70 [82]	2.28 (0.28); 2.20 (0.30); 1.90–2.60 [3]
Postoperative CRP—mean (SD); median (IQR); range [missing]	11.1 (5.9); 9.5 (7.3); 1.5–31.2 [44]	10.9 (5.7); 9.3 (6.9); 1.5–31.2 [44]	14.0 (7.6); 11.4 (11.4); 6.4–26.1 [0]
Clinical and operative characteristics			
ASA score, *n* (%)—I/II/III/IV	4 (2.6)/70 (46)/68 (44)/11 (7.2)	4 (2.8)/66 (46)/65 (45)/10 (6.9)	0 (0)/4 (50)/3 (38)/1 (13)
Surgical approach, *n* (%)—laparoscopic/open	27 (18)/126 (82)	26 (18)/119 (82)	1 (13)/7 (88)
Anastomosis type, *n* (%)—colo-colic/colorectal	63 (41)/90 (59)	57 (39)/88 (61)	6 (75)/2 (25)
Anastomotic configuration, *n (*%)—side-to-side/side-to-end or end-to-side/end-to-end	17 (11)/37 (24)/99 (65)	16 (11)/36 (25)/93 (64)	1 (13)/1 (13)/6 (75)
Operative duration, min—mean (SD); median (IQR); range	225 (71); 225 (90); 75–465	227 (71); 225 (90); 75–465	189 (45); 190 (39); 120–270
Postoperative length of stay, days—mean (SD); median (IQR); range	11 (7); 9 (3); 3–60	10 (5); 8 (3); 3–39	31 (13); 29 (3); 18–60
Mortality *, n (%)—no/yes	149 (97)/4 (3)	144 (99)/1 (1)	5 (63)/3 (38)

Abbreviations: AL, anastomotic leakage; ASA, American Society of Anesthesiologists; CRP, C-reactive protein; IQR, interquartile range; SD, standard deviation; NLR, neutrophil-to-lymphocyte ratio; R0, microscopically negative resection margin; R1, microscopically positive resection margin; *, thirty-day postoperative mortality. Data are presented as *n* (%) or descriptive statistics, as indicated. Percentages for variables with missing data are based on available observations. Numbers in square brackets indicate missing values. **, large bowel obstruction: presence of clinical signs and radiological findings consistent with intestinal obstruction (accounting for 31/166 patients with stenosing tumours).

**Table 5:** Univariate analysis of factors associated with anastomotic leakage in patients treated with a primary anastomosis (N = 153).

Predictor	N	AL, *n*	OR (95% CI)	*p*-Value
Preoperative haematological and inflammatory parameters				
Haemoglobin	152	8	0.66 (0.46–0.92)	0.018
Anaemia (yes vs. no)	152	8	3.13 (0.74–15.8)	0.13
WBC	152	8	1.07 (0.79–1.35)	0.60
Leucocytosis (yes vs. no)	152	8	3.67 (0.50–18.1)	0.14
Platelets	152	8	1.00 (1.00–1.01)	0.16
Neutrophils	152	8	1.13 (0.80–1.46)	0.41
Lymphocytes	152	8	0.51 (0.11–1.69)	0.32
NLR	152	8	1.23 (0.85–1.68)	0.22
PLR	152	8	1.00 (1.00–1.01)	0.16
Fibrinogen	150	8	1.10 (0.41–2.32)	0.82
Postoperative laboratory parameters				
Total protein	63	6	0.53 (0.19–1.20)	0.16
Albumin	68	5	0.03 (0.001–0.27)	0.011
CRP	109	8	1.08 (0.96–1.20)	0.17
Pathological characteristics				
Lymph node yield	150	8	0.86 (0.74–0.98)	0.038
LVI (yes vs. no)	134	6	0.81 (0.11–4.29)	0.81
Peritumoral inflammatory infiltrate (yes vs. no)	100	5	0.27 (0.03–5.71)	0.27
Tumour budding (yes vs. no)	85	2	0.20 (0.01–5.33)	0.27
Tumour size	152	8	1.06 (0.74–1.46)	0.72
Stenosing tumour (yes vs. no)	152	8	4.08 (0.70–77.3)	0.19
ASA score and surgical characteristics				
ASA 1–2	74	4	Reference	—
ASA 3	68	3	0.81 (0.15–3.80)	0.79
ASA 4	11	1	1.75 (0.08–13.4)	0.63
Laparoscopic approach	27	1	Reference	—
Open approach	126	7	1.53 (0.26–29.2)	0.70
Colo-colic anastomosis	63	6	Reference	—
Colorectal anastomosis	90	2	0.22 (0.03–0.97)	0.044
Side-to-side anastomosis	17	1	Reference	—
Side-to-end/end-to-side anastomosis	37	1	0.44 (0.02–11.7)	0.57
End-to-end anastomosis	99	6	1.03 (0.16–20.2)	0.98
Operative duration	153	8	0.99 (0.98–1.00)	0.15

Abbreviations: AL, anastomotic leakage; ASA, American Society of Anesthesiologists; CI, confidence interval; CRP, C-reactive protein; LVI, lymphovascular invasion; NLR, neutrophil-to-lymphocyte ratio; OR, odds ratio; PLR, platelet-to-lymphocyte ratio; WBC, white blood cell count.

## Data Availability

The patients’ data were obtained from the medical documents of the Bucharest Oncological Institute and cannot be made publicly available as they contain personal and confidential data, but any information about these documents can be obtained upon reasonable request from the corresponding author.
